# Breast milk-derived human milk oligosaccharides promote *Bifidobacterium* interactions within a single ecosystem

**DOI:** 10.1038/s41396-019-0553-2

**Published:** 2019-11-18

**Authors:** Melissa A. E. Lawson, Ian J. O’Neill, Magdalena Kujawska, Sree Gowrinadh Javvadi, Anisha Wijeyesekera, Zak Flegg, Lisa Chalklen, Lindsay J. Hall

**Affiliations:** 1 Gut Microbes & Health, Quadram Institute Bioscience, Norwich Research Park, Norwich, UK; 2 Department of Food & Nutritional Sciences, University of Reading, Reading, UK; 3 Present Address: Lydia Becker Institute for Immunology and Inflammation & Wellcome Trust Centre for Cell Matrix Research, Manchester Academic Health Science Centre, University of Manchester, Manchester, UK; 4 Present Address: APC Microbiome Ireland, University College Cork, Biosciences Building, Cork, Ireland

**Keywords:** Microbiome, Bacterial physiology, Microbial ecology

## Abstract

Diet-microbe interactions play an important role in modulating the early-life microbiota, with *Bifidobacterium* strains and species dominating the gut of breast-fed infants. Here, we sought to explore how infant diet drives distinct bifidobacterial community composition and dynamics within individual infant ecosystems. Genomic characterisation of 19 strains isolated from breast-fed infants revealed a diverse genomic architecture enriched in carbohydrate metabolism genes, which was distinct to each strain, but collectively formed a pangenome across infants. Presence of gene clusters implicated in digestion of human milk oligosaccharides (HMOs) varied between species, with growth studies indicating that within single infants there were differences in the ability to utilise 2′FL and LNnT HMOs between strains. Cross-feeding experiments were performed with HMO degraders and non-HMO users (using spent or ‘conditioned’ media and direct co-culture). Further ^1^H-NMR analysis identified fucose, galactose, acetate, and N-acetylglucosamine as key by-products of HMO metabolism; as demonstrated by modest growth of non-HMO users on spend media from HMO metabolism. These experiments indicate how HMO metabolism permits the sharing of resources to maximise nutrient consumption from the diet and highlights the cooperative nature of bifidobacterial strains and their role as ‘foundation’ species in the infant ecosystem. The intra- and inter-infant bifidobacterial community behaviour may contribute to the diversity and dominance of *Bifidobacterium* in early life and suggests avenues for future development of new diet and microbiota-based therapies to promote infant health.

## Introduction

The early-life developmental window represents a critical time for microbe–host interactions as this is when foundations for future health and well-being are established. Colonisation of pioneer microbes shortly after birth represents a key first step in this mutualistic relationship; shaping the developing microbial community, and in turn impacting numerous host physiological processes [1–5]. Although the microbiota of adults is complex in nature, the gastrointestinal (GI) tract of full-term healthy infants is relatively simplistic, dominated by the genus *Bifidobacterium* that can persist into early childhood [3, 6]. In the first months of birth, the loss of *Bifidobacterium* species or gain of other bacteria during this critical window of opportunity, may significantly alter the ‘natural’ progression of the microbial community that may lead to a variety of negative consequences for host health including a predisposition to autoimmune/metabolic diseases (like allergies and childhood obesity) [1, 2].

Due to the high abundance of *Bifidobacterium* within the infant gut, this genus can be considered a foundation microbiota member that strongly influences the intestinal environment, the structure of burgeoning microbial communities in early life, and ultimately host development [7, 8]. Infant diet is suggested to be one of the key factors that shapes the early-life microbiota, and breast-feeding encourages *Bifidobacterium* growth within the infant gut, thus highlighting a strong diet-microbe association [9]. Recently the WHO and the Scientific Advisory Committee on Nutrition (UK) released new guidelines regarding the optimal time to start breast feeding and highlighted the health benefits associated with solely breast-feeding infants [9, 10]. Indeed, breast-fed and formula-fed infants differ in microbial composition [11], including significant differences in bifidobacterial populations, which has also been linked to differential health outcomes e.g., induction of allergies, asthma, and obesity in formula-fed infants [11, 12]. Breast milk contains prebiotic human milk oligosaccharides (HMOs) that preferentially feed beneficial gut bacteria, including *Bifidobacterium* [13]. HMOs are unconjugated glycans with a lactose core varying in chain length from 3 to 15 carbohydrates (glucose, galactose, fucose, N-acetylglucosamine (GlcNAc), and N-acetylneuraminic acid (NeuAc) or sialic acid) [14, 15]. The lactose HMO backbone can additionally be fucosylated or sialylated to form trisaccharide HMO structures, termed 2′ or 3′-fucosyllactose (2′or and 3′ or 6′-sialyllactose (3′ or 6′SL), respectively (reviewed in [15]). The variety of HMO appears endless; to date over 200 different structures of HMOs have been identified in breast milk [15].

Establishment of this breast milk associated bifidobacterial dominant community is aided, in part, by the abundance of carbohydrate utilisation genes [16, 17], and specific gene clusters allowing for metabolism of HMOs [18–20], which are absent in many adult associated strains [13]. Notably, previous work has indicated that multiple *Bifidobacterium* strains coexist in a single infant GI tract, rather than one strain dominating and competitively excluding all other strains [21]. To investigate these key community dynamic questions, we have probed the genomic and phenotypic similarities between bifidobacterial strains that coexist in the same individual, including their responses to specific early-life diet components, namely HMOs. By examining microbial interactions on a strain-level we provide important insights into how multiple species of *Bifidobacterium* coexist within a single infant in early life, which may have implications for design of diet- and microbial-based early-life therapies.

## Results

### *Bifidobacterium* dominate the gut microbiota of breast-fed infants

**Fig. 1:**
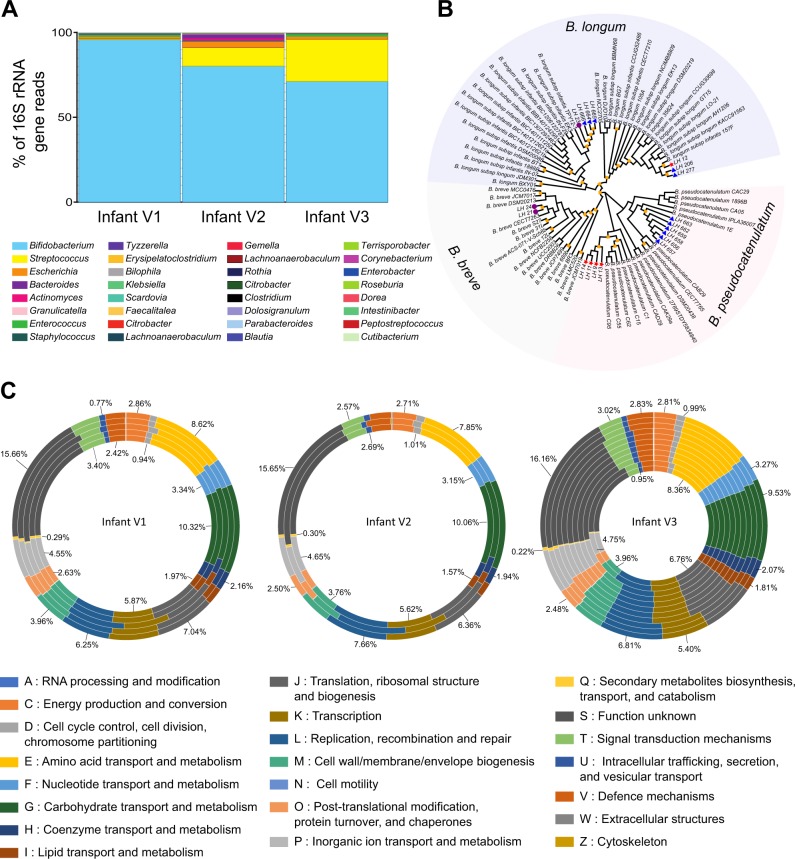
**a** Faecal bacterial community profiles of three healthy, full-term infants as determined by 16S rRNA gene sequencing. Paired-end reads were generated using the MiSeq Illumina platform, all data sets were normalised and relative abundance of each bacterial taxa is represented in percentages of number of total reads for the top ten most prevalent genus in each infant. Bar colours represent different genus taxa, and bar lengths signify the relative abundance of each taxon. 16S rRNA bacterial profiles are named according to the sample used for bifidobacteria isolation. V1 at 102 days of age, V2 at 174 days of age, and V3 at 159 days of age. The number of reads obtained by 16S rRNA gene sequencing data for each sample can be found in Table S2. **b** Core-genome phylogeny of 83 *Bifidobacterium* isolates, 19 of which are novel strains identified in this study and denoted by arrows and the annotation of LHXX. Isolates from infant V1 are denoted with a red star, V2 with a purple circle, and V3 with a blue triangle. Bootstrap values >70 are shown with a yellow square on each node. **c** ORFs from each genome was submitted to eggNOG-mapper (http://eggnogdb.embl.de/#/app/emapper) for functional classification. The proportion of ORFs for each classification was calculated and is presented as a percentage of total ORFs in each genome. The values indicated for each orthologous group represent the average percentage of ORFs in that group for all genomes in that infant

To investigate bifidobacterial community interactions in early life, the faecal microbial community profiles from three full term, healthy infants (herein termed infant V1, V2, and V3) were subjected to metataxonomic profiling using 16S rRNA gene sequencing (Figs. 1a, S1A and Tables S1 and S2). At the time of sample collection, all infants were of similar age (mean = 145 ± 38d), born vaginally and exclusively breast-fed (two isolates from infant V3 where isolated from an earlier stool sample, Fig. S1B). In agreement with previous studies [22–24], we observed a high prevalence of *Bifidobacterium* in each infant faecal sample (mean = 82.53 ± 12.36%). Further analysis indicated a dynamic bifidobacterial community comprised of different strains and species and as such we chose multiple *Bifidobacterium* strains to explore how these genus-specific microbial community interact with each other. Strains were next examined for probiotic-traits (‘live microorganisms which when administered in adequate amounts confer a health benefit on the host’ [25]); these traits include (but are not limited to) the ability to survive in aerobic conditions, bile acids, and after acid shock (pH2 akin to stomach acid; Fig. S2). Briefly, we found that LH23 from infant V2 was able to withstand exposure to 0.3% bile salt when compared to other strains tested (Fig. S2A). Examining isolate response against specific bile acids including the hydrolysis of taurocholic, taurodeoxycholic, and sodium glycodeoxycholate bile acids demonstrated variability in strain responses. Strain LH12 from infant V1 could only use the taurodeoxycholic bile acid; whilst isolates from the other infants, such as LH206 and LH277 from V3 could not hydrolyse any of the bile salts tested regardless of the fact these strains both possess a known bile salt hydrolase gene (Tables S3 and S4). We also found high variability between the survival ability of strains from the same infants (and within the same species, *species annotation described below*) when tested after against oxygen exposure and acid shock. In total, 18 of 19 strains showed resistance to all the above environmental stressors, properties that are advantageous when choosing strains for use as probiotics [25].

As the phenotypic tests suggested there may be strain-level differences, we sequenced and performed comparative genomic analysis on all novel 19 bifidobacterial strains. Sequencing and assembly using the PROKKA pipeline resulted in sets of contigs ranging from 7 to 46 per strain (Table S1). The G+C content ranged from 56.50% for LH9 to 60.04% for LH277, while the number of predicted ORFs was lowest in LH11 (1888), and highest in LH23 (2521) (Table S1). Genome sizes ranged from 2.25 Mb (LH13) to 2.75 Mb (LH23), with an average of 2.38 Mb, consistent with previously published data [26]. Genetic relatedness based on core-genome phylogeny (Fig. 1b) and average nucleotide identity (ANI) values (Fig. S3), indicates clustering into three main phylogenetic groups; *Bifidobacterium longum* (encompassing the members of the *longum* and the *infantis* subspecies), *Bifidobacterium breve* and *Bifidobacterium pseudocatenulatum* groups. Strains isolated from infant V1 were classified as either *B. longum* subspecies *longum* (hereafter referred to as *B. longum*, LH12) or *B. pseudocatenulatum* (LH9, LH11, LH13, LH14); V2 strains classified as *B. breve* (LH21, LH24) or *B. longum* subspecies *infantis* (hereafter referred to as *B. infantis*, LH23), and V3 strains were classified as either *B. pseudocatenulatum* (LH656, LH657, LH658, LH659, LH662, LH663), *B. infantis* (LH664, LH665, LH666), or *B. longum* (LH206, LH277) (Fig. 1b).

### Functional annotation of genomes of *Bifidobacterium* from infants—carbohydrate utilisation

We next functionally characterised the open reading frames of each genome with EggNOG-mapper. This identified carbohydrate transport and metabolism as the second most abundant genes present in all genomes, linking to the glycan rich environment of the colon [27, 28] (the most annotated gene function was: unknown function; Fig. 1c and Table S5A). V1 strains had the largest proportion of carbohydrate metabolism and transport genes (10.32%), whilst strains from infant V2 and V3 were slightly lower (10.06% and 9.53% respectively). Intraspecies gene differences in carbohydrate metabolism have been well described for *B. pseudocatenulatum* (albeit limited), and more so for *B. longum* and *B. breve* [29–31]. The *B. pseudocatenulatum* genome has a high proportion of glycosyl hydrolase family (GH)-43 enzymes that aid in the degradation of complex plant glycans, similar to other species including *B. dentium* and *Bifidobacterium adolescentis* [32]. As we isolated a number of *B. pseudocatenulatum* strains from a single infant—consistent with other studies [31]—the ROARY pipeline was used to identify unique genes in the pangenome from infants V1—333 unique genes (215 functionally assigned)—and V3—333 unique genes (236 functionally assigned, Table S6A). Many of these genes were annotated as encoding proteins involved in carbohydrate transport and metabolism (Table S5B). Further *B. pseudocatenulatum* investigation (Fig. S4A, Tables S6B and S6C) indicated strains from the same infant shared many core genes (Infant V1 = 1779 genes; Infant V3 = 1839 genes), with differences in accessory genes (Infant V1 = 108 genes, Infant V3 = 83 genes, Fig. S4). Notable characterisation findings indicated V1 strains LH13 and LH14 possess unique putative carbohydrate utilisation gene clusters; LH13 encodes a beta-xylosidase, ABC transporters and multiple permeases (LH_13_00067-LH_13_00071), while the LH14 cluster encodes a beta-D-glucosidase, alpha-xylosidase, two permeases and a putative sugar-binding periplasmic protein precursor (LH_14_01835-LH_14_01839) which suggests this strain may perform xylan degradation (Fig. S4A). LH14 genome also encodes fimbriae and sortase genes implying the presence of sortase dependent fimbriae (Fig. S4A). LH13, LH656 and LH658 have partial but incomplete prophage clusters within their genomes (Tables S7A and S7B). However, due to the draft nature of the genomes analysed, and regardless of differences in the accessory genome (discussed above and in Tables S7A and S7B), isolates of the same species, from the same infant, at the same time point may in fact be clonal strains of each other rather than independent isolates.

**Fig. 2:**
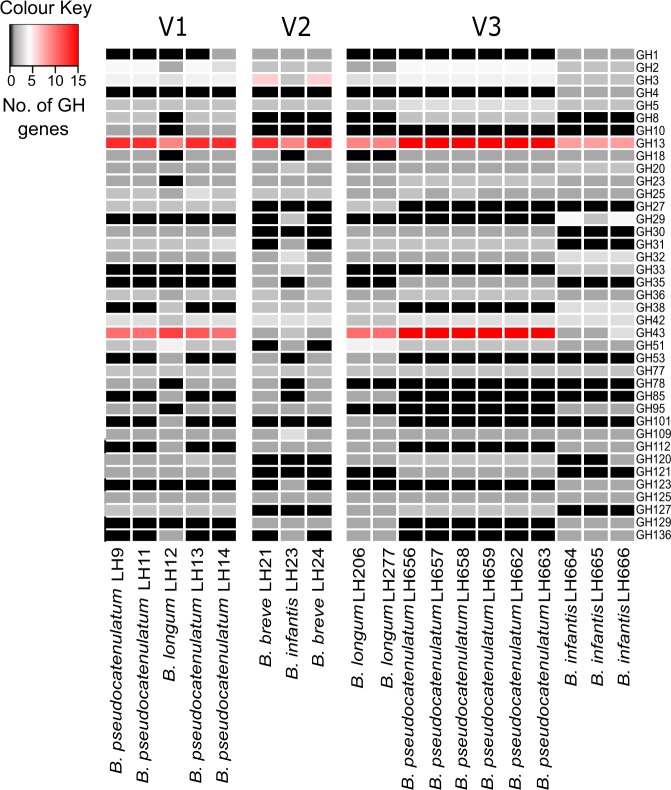
Functional classification of *Bifidobacterium* genomes. Presence of genes encoding glycosyl hydrolases was determined using the dbCAN server (http://csbl.bmb.uga.edu/dbCAN/) which annotates genes based on HMMs of GH generated from data in the CAZy database (http://www.cazy.org). The heatmap shows the number of ORFs annotated as GH for each GH family (*y*-axis) for each genome (*x*-axis) (Enumeration of GH ORFs in subfamilies of GH5, GH13 and GH43 is shown in Supplementary Fig. 4B)

As *Bifidobacterium* possess a large repertoire of glycoside hydrolases (GH) that facilitate digestion and metabolism of glycans in the gut we analysed and compared GH repertoires (Figs. 2 and S4B). A total of 39 different GH families were found in all *Bifidobacterium* strains isolated; ~62 GH genes (3.68 % of OFRs) in *B. pseudocatenulatum* strains, followed by ~48 GH genes (2.82% of ORFs) per *B. longum* genome, 46 GH genes (2.51 % of ORFs) per *B. breve* genome, and finally 42 GH genes (2.11% of ORFs) per *B. infantis* genome, consistent with published data [27].

The predominant GH in all strains was GH13 which represents enzymes for the hydrolysis of alpha-glucosidic linkages in plant di-, oligo-, and polysaccharides [29]. Whilst, the second most abundant GH family present in infant V1 and V3 (but not infant V2) was GH43, which contains enzymes like beta-xylosidases (involved in xylan digestion). The presence of GH33 gene cluster that encodes exo-sialidases in *B. infantis* and *B. brev*e strains from infants V2 and V3, suggests these strains may be capable of directly utilising host mucins to liberate sialic acids, as well as metabolise free sialic acids in the gut (for potential cross-feeding, [33] (Fig. 2). However, the absence of this GH gene (in some *B. pseudocatenulatum* and *B. longum* strains) does not strictly indicate a lack of cross-feeding of sialic acids [34]. Other highly represented GH families within *Bifidobacterium* populations identified were GH3, GH2, and GH42. GH3 family members include beta-glucosidases that hydrolyse a wide variety of glycans present in plant cell walls; while GH2 and GH42 family members contain beta-galactosidases that are active on galactooligosaccharides and galactans found in plant cell walls, but can also metabolise lactose, the primary sugar in breast milk.

### Functional annotation of genomes of *Bifidobacterium* from infants—human milk oligosaccharide utilisation

Many early life-associated bifidobacterial species and strains contain GH genes that are specifically target HMOs for degradation and metabolism [35]. *B. infantis*, *B. breve*, *B. longum*, and *B. pseudocatenulatum* have genomic clusters required for HMO utilisation [18, 19, 35, 36], thus we searched for the presence of these clusters in our novel 19 strains. Genomically *B. infantis* ATCC 15,697 has a large 45 kb HMO cluster (BLON_RS12070-BLON_RS12215) that allows for digestion of multiple HMOs [18]. In our strains, we identified homologous HMO clusters in *B. infantis* LH23, LH664, LH665, and LH666 (Fig. S5A); although our strains often had altered gene cluster organisation to the published cluster (Fig. S5A) potentially due to incomplete genome information.

**Fig. 3:**
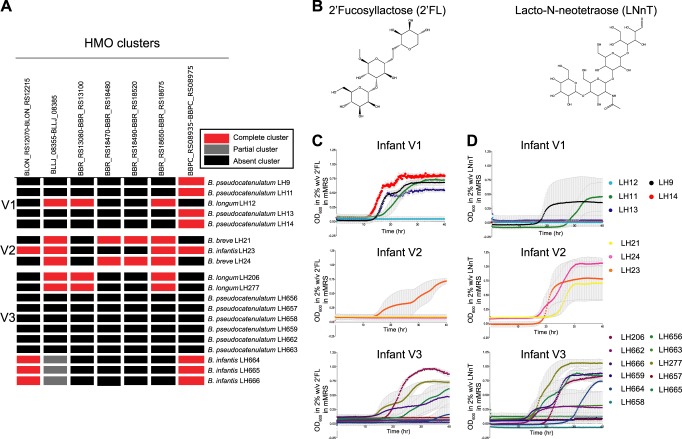
HMOs function as key carbon sources for *Bifidobacterium* growth. **a** HMO heatmap illustrating the presence of known HMO gene clusters in the 19 novel infant isolates (see “Methods” section for details); **b** chemical structure of HMO 2′FL and LNnT generated by ChemDraw; **c** Growth kinetics of all 19 strains in mMRS with either HMO 2′FL or **d** LNnT as a sole carbon source; all strains from each infant are illustrated together. Data shown are a representative graph of three independent experimental repeats, containing the mean from duplicate/triplicate well measurements

A specific gene cluster has also been identified for 2′FL (fucosyllated HMO) in *B. longum* SC569, which contains an alpha1-3/4-fucosidase (GH29) and/or alpha1-2 fucosidase (GH95) within a carbohydrate utilisation gene cluster (BLNG_01254-BLNG_01264) [36]. We could not identify any homologous gene clusters or any potential alpha-fucosidases in our *B. longum* strains; but *B. infantis* (LH23, LH664, LH665, and LH666) strains from infant V2 and V3 did have these encoded (data not shown). In addition, a similar 2′FL gene cluster in *B. pseudocatenulatum* type strain DSM 20438, containing a single alpha-fucosidase (GH95) as previously reported [36], was also identified in *B. pseudocatenulatum* strains from infant V1: LH9, LH11, LH13, and LH14, including the key GH95 gene (Fig. 3a).

LNT and LNnT HMO clusters have been well annotated in *B. breve* UCC2003; *lnt* cluster (BBR_RS13080-BBR_RS13100); *lac* cluster (BBR_RS18470-BBR_RS18480), the *nah* cluster (BBR_RS18490-BBR_RS18520) and *lnp/glt* cluster (BBR_RS18650-BBR_RS18675) [19]. Homologues to the *lac, nah*, and *lnp/lgt* clusters were identified in *B. breve* LH21 and LH24. The *lnp/glt* cluster was also identified in *B. longum* strains LH12, LH206, and LH277 [37]. Interestingly, the *lnt* HMO cluster was not present in *B. breve* LH21 and LH24; however, we could identify homologous clusters in *B. infantis* strains LH206, LH277, and *B. longum* strain LH12. It should be noted that these are draft whole genome sequences and thus, it is possible that the absence of the *lnt* cluster in LH21 and LH24 may be due to this.

HMOs that are sialylated require sialidase enzymes for degradation such as the extracellular sialidase (SiaBb1) found in *B. bifidum* ATCC 15696 that permits sialylated HMOs digestion on the bacterial cell surface [38, 39]. Our analysis identified sialidases in *B. infantis* strains LH23, LH664, LH665, and LH666; however, in all strains the transmembrane domains was absent suggesting that these strains may perform intracellular digestion of sialylated HMOs (Table S8), as previously shown for *B. infantis* ﻿ATCC 15697 [18].

The presence of different HMO utilisation clusters in species within a single infant further highlights the adaptation of *Bifidobacterium* to a diet rich in HMO and potentially facilitating establishment within the early-life gut.

### Phenotypic characterisation of HMO usage

Next to lactose, HMOs are the second most abundant carbohydrate in breast milk (5–15 g/L in breast milk) [40–42]. Although we identified putative HMO genomic clusters in our strains, we next sought to investigate the ability of our strains to use HMOs as a sole carbon source for growth; 2′FL and LNnT (Fig. 3b–d). We identified 9 strains capable of using 2′FL and 12 strains capable of using LNnT (or both) from each infant. In agreement with genomic analysis indicating that V1 *B. pseudocatenulatum* strains (LH9, LH11, LH13, and LH14) contained a known fucosylated HMO utilisation gene cluster, we also observed growth on 2′FL [36] (Fig. 3a–c). The *B. longum* LH12 strain from infant V1 did not contain a 2′FL gene cluster and was unable to use this HMO for growth. Testing strains from infant V1 for growth with the HMO LNnT indicated that only *B. pseudocatenulatum* LH11 could use the more complex HMO, despite lacking known enzymatic clusters. *B. infantis* LH23 isolated from infant V2 could degrade both 2′FL and LNnT and contains key GH genes previously associated with the metabolism of these HMOs [18, 43]. Neither *B. breve* strains LH21 or LH24 grew on 2′FL potentially as they lack a GH29 fucosidase gene [46]. All three strains (LH21, LH23, and LH24) grew well with LNnT as a sole carbon source (Fig. 3d). The distinct genomic cluster implicated in LNnT metabolism in *B. breve* UCC2003 was not identified in LH24, but this may be due to the draft nature of the genome. The bifidobacterial community within infant V3, containing the largest group of isolates, however none of the *B. pseudocatenulatum* strains could use either HMOs tested (possibly linking to absence of appropriate encoded HMO clusters). All *B. longum* and *B. infantis* strains metabolised both HMOs tested (although there is variability in growth kinetics for each strain), supporting previous studies and our genomic analysis indicating *B. infantis* can grow on a wide range of HMOs, and some *B. longum* strains can also metabolise 2FL and LNnT [18, 30, 36, 43]. When HMO utilisation was tested in type strains for *B. longum, B. infantis*, and *B. pseudocatenulatum* we found there was not a global ability of all strains within a species of *Bifidobacterium* to utilise HMOs (Fig. S5B), demonstrating that HMO utilisation is dependent on the type of HMO and the strain (rather than the species) tested.

### HMOs degradation by *Bifidobacterium* influences growth dynamics of neighbouring strains

**Fig. 4:**
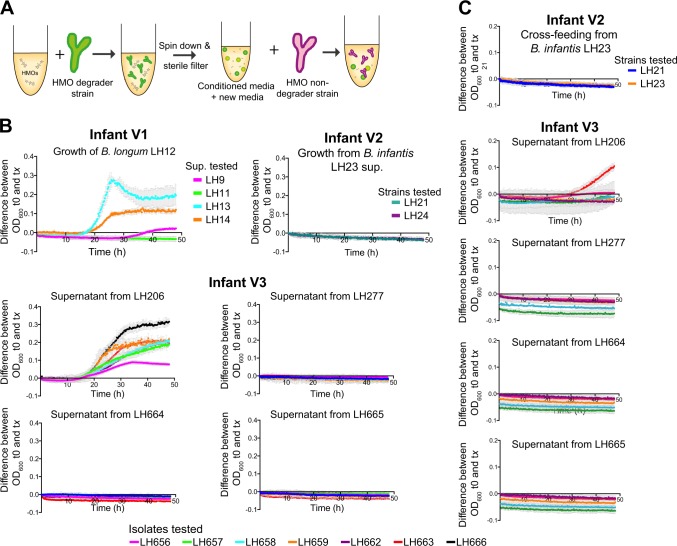
Cross-feeding networks within *Bifidobacterium* communities. **a** Schematic illustrating experimental set-up and preparation of conditioned media for cross-feeding experiments. **b** Using conditioned media from strains capable of using either HMO 2′FL or **c** LNnT was combined with fresh mMRS (1:1) and used as growth media for all HMO nondegrader isolates within the same ecological niche. Representative graph shown from three independent experimental repeats, each point represents the mean ± standard deviation

Previous work, including data presented in this study, indicates that multiple *Bifidobacterium* species and strains exist as a community within a single microbial (i.e., infant) ecosystem [23, 24]. To address if infant-specific strains modulate growth of neighbouring strains we first generated spent or ‘conditioned’ media from identified ‘HMO-degraders’ strains (Fig. 4a; HMOs 2′FL; Fig. 4b, or LNnT; Fig. 4c. This conditioned media were then used as a growth substrate for other ‘non-HMO users’ identified within the same microbial (infant) community (Fig. 4a). 2′FL derived-substrates from all *B. pseudocatenulatum* strains in infant V1 (LH9, LH11, LH13, LH14) supported growth of their ‘non-HMO user’ *B. longum* LH12 (Fig. 4b), indicating potential cross-feeding. Conversely, infant V2-associated *B. infantis* LH23 2′FL HMO degradation metabolic by-products did not suppport *B. breve* (LH21 and LH24) growth. Infant V3 *B. longum* and two *B. infantis* isolates (LH206, LH277, LH664, and LH665 respectively) grew on 2′FL (Fig. 3c). However, only conditioned media from isolate *B. longum* LH206 were able to support the growth of other isolates within the same infant (Fig. 4b), even though bioinformatic analysis did not identify any alpha-fucosidase genes in LH206. Moreover, LH206-2′FL conditioned media enhanced the growth of all tested strains (*B. infantis* and *B. pseudocatenulatum)*, which suggests that metabolism of 2′FL by LH206 may generate a wider variety of growth-promoting components. To confirm conditioned media findings, we also monitored bacterial DNA concentration (as an indicator of abundance) over time with quantitative PCR. Culturing of *B. pseudocatenulatum* LH13 together with *B. longum* LH12 on 2′FL and showed growth of both strains in co-culture (Fig. S5C). We also observed growth of both strains when either *B. pseudocatenulatum* strains LH657, LH659, or LH663 were co-cultured with *B. longum* LH206 on 2′FL (Fig. S5C).

Overall fewer isolates appeared to utilise LNnT, likely because LNnT is a structurally more complex HMO that contains a glycosidic linkage within the nonreducing terminal disaccharide, Galβ1-3/4GlcNAc [44]. LH11 from infant V1 grew in the presence of LNnT; however, the metabolic by-products did not support growth of any other identified strains in this infant. *B. infantis* LH23 strain from infant V2 and *B. infantis* LH664 and LH665 from infant V3 utilised LNnT, but we did not detect the presence of cross-feeding (Fig. 4c). Both *B. longum* LH206 and LH277 utilised LNnT, however only metabolites produced following LNnT utilisation in the conditioned media from the strain LH206 supported the growth of other ‘non-HMO users’ in infant V3. We observed moderate growth for specific strains *B. pseudocatenulatum* LH656, LH657, and LH663.

### HMO degradation by *Bifidobacterium* and associated metabolites

**Fig. 5:**
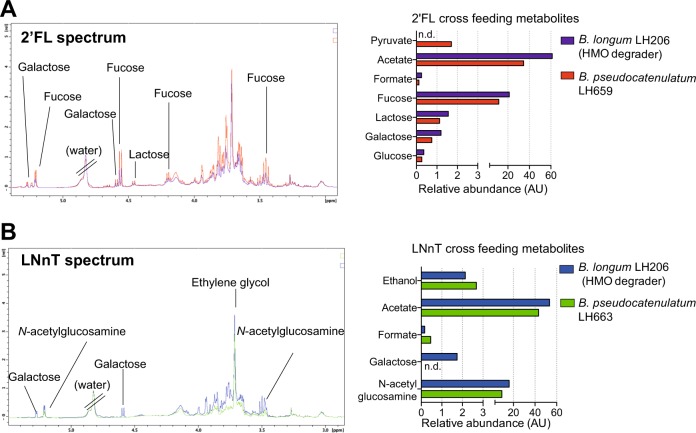
Identification of cross-feeding metabolites. **a** ^1^H-NMR spectrum to identify metabolites involved in cross-feeding between 2′FL-degrader *B. longum* LH206 and nondegrader *B. pseudocatenulatum* LH659 and semi-quantification of identified metabolites. **b** ^1^H-NMR spectrum to identify metabolites involved in cross-feeding between *LNnT* degrader *B. longum* LH206 and nondegrader *B. pseudocatenulatum* LH657 and semi-quantification of identified metabolites. n.d indicates metabolites were not detected

To explore potential HMO degradation products, we used ^1^H-NMR to identify metabolic compounds in cross-feeding studies. *B. longum* LH206 generated acetate and formate after 2-FL metabolism (formate is an intermediate metabolite of fermentation to lactate) [45] (Fig. 5a). Metabolism of 2′FL by LH206 (with concurrent reduction in 2′FL) was followed by an increase in fucose and, to a lesser extent, lactose suggesting 2′FL is cleaved into fucose and lactose by this strain (Table S9). The high levels of fucose remaining in the media following growth suggests that fucose is not fully metabolised by this strain. Fucose was not detected following *B. pseudocatenulatum* LH13 growth in 2′FL but 1, 2-propanediol was present suggesting that this strain metabolises fucose to 1, 2-propanediol as described elsewhere [46] (Fig. S7 and Table S9).

Cell free supernatant from the growth of LH206 on 2′FL was diluted (1:1) with fresh 2′FL-free media and used as media to grow LH659, as described above (Fig. 4a). When this dilution is taken into account modest increases in acetate (30.35–34.64 R.U) were detected, suggesting low level growth of LH659 (Fig. S6 and Table S9). We observed a reduction in 2′FL and production of acetate, ethanol, formate, and 1,2 propandiol by HMO degrader *B. pseudocatenulatum* LH13. Growth on LH13 supernatant by the HMO nondegrader *B. longum* LH12 (infant V1) produced low levels of acetate, ethanol, and pyruvate indicating modest metabolic activity by LH12 under these conditions. (Fig. S7 and Table S9).

We also investigated metabolites from LNnT degradation by *B. longum* LH206, and subsequently profiled metabolites involved with cross-feeding to the non-LNnT degrader, *B. pseudocatenulatum* LH663 (Figs. 5b, S8 and Table S9). From LH206-LNnT digestion there were increases in SCFA acetate, the energy-related compound formate, and the end product of fermentation ethanol, suggesting LNnT degradation by LH206. In addition, galactose was liberated by LH206 into the supernatant, and after LH663 growth it could no longer be detected. Growth of LH663 in LH206 supernatant also resulted in an increase in acetate, ethanol, and formate, collectively this data suggests LH663 was metabolically active and growing in the conditioned media from LH206 (Table S9). Similar results were found for cross-feeding of LH206 to LH657 (Fig. S9 and Table S9). These data suggest that metabolites resulting from HMO metabolism by one species allow for modest growth of another, non-HMO metabolising, species.

## Discussion

*Bifidobacterium* spp. are central players in the early-life microbiota and healthy infant development. We show that this genus is present at very high levels in breast-fed infants, and that distinct bifidobacterial communities exist within an individual infant, consistent with other findings [47]. Our data indicate differences in genomic content for these individual strains, which links to their ability to thrive on breast milk-associated dietary components like HMOs by multiple members of *Bifidobacterium* within a single infant (‘community’). These results further highlight the role of bifidobacterial communities in adaption to a breast milk (HMO) diet.

We isolated a significant number of diverse bifidobacterial strains and species including members commonly associated with infants including *B. infantis, B. longum, B. breve*, and *B. pseudocatenulatum* from three healthy, breast-fed infants [48, 49]. We identified genotypic and phenotypic differences between these strains and species within a single infant bifidobacterial community. However, these differences appear to be complementary and likely contributing to a flexible and cooperative relationship between the infant breast milk diet (i.e., HMOs) and the early-life *Bifidobacterium* populations. HMOs represent a key nutritional component of breast milk, but these complex carbohydrates cannot be directly metabolised by the infant [50], but rather specialised members of the resident gut microbiota breakdown these HMOs. In particular certain species and strains of *Bifidobacterium* utilise HMOs and likely contributes to their ability to function as a foundation genus within the wider context of the early-life microbiota [15, 41]. Multiple studies have identified genomic clusters for the degradation of milk carbohydrates, including specific clusters for utilisation of specific HMOs [18, 19, 36]. We identified that the genomic arrangement of these clusters exhibits interspecies variability and consistent with other studies, the presence of gene members from these HMO clusters does not always result in a growth phenotype on the specified HMOs. For instance, both *B. breve* strains in this study possessed a key GH for fucosylated HMO degradation but did not grow on 2′FL which could potentially be due to the lack of a second fucosidase (GH29) or appropriate transport genes [46]. Furthermore, we identified growth on HMOs in strains lacking known clusters, suggesting a wider variety of novel gene clusters devoted to HMO degradation that could be explored further to provide more mechanistic rationale for development of early-life microbiota therapies.

The use of key metabolites produced from HMO degradation from one species of *Bifidobacterium* to another, highlights a potential way to permit growth of multiple different bifidobacterial species and strains within the breast-fed infant gut [13, 27, 38]. Moreover, a cooperative balance between bifidobacterial strains in the early-life microbiota [51] may further enhance their dominance in breast-fed infants by enabling a genus-specific exploitative competition i.e., depleting the GI tract of breast milk-derived nutrients, thereby preventing colonisation of other microbes, including pathobionts. Our direct co-cultures studies suggest there is growth enhancement of non-HMO degraders in the presence of a corresponding HMO-utiliser. Conditioned media studies also suggest that metabolites from 2′FL and LNnT degradation may promote (low level) growth of nondegraders *Bifidobacterium* species within the same infant, indicating sharing of resources. For example, metabolites from 2′FL degradation by *B. pseudocatenulatum* strains appears to support growth of *B. longum*, a process that has not previously been described for these species. Quantified metabolic products indicates that fucose, acetate, pyruvate, and 1,2-propanediol are liberated from HMO degradation and could potentially function as candidates for cross-feeding. However, we did not see evidence of metabolism of these compounds by the *B. longum* strain as has been reported with other members of the infant microbiota; such as the 2′FL metabolic end product 1,2-propanediol which drives cross-feeding interactions between *B. infantis* and *Eubacterium halli* [52]. Recent work suggests that extracellular sialidases are the main source of cross-feeding interactions between bifidobacterial strains, as has been described for *B. longum*, which by producing sialylated carbohydrates and free sialic acid promotes *B. breve* growth [38, 39]. Currently there is little evidence suggesting cooperation between non-extracellular HMO degraders. Whilst we have not detected known extracellular sialidases (within HMO-degrading putative cross-feeding strains), it may be our strain(s) encode novel enzymatic clusters that perform this type of extracellular degradation. Moreover, intracellular HMO utilisation clusters may also be important in cross-feeding with metabolites released via cell lysis (i.e., metabolites leakage [53]) or in some cases actively secreted [54], as has recently been proposed with genome-scale metabolic models (including ‘costless’ secretion of amino acids by *B. adolescentis* [55]). As we utilised NMR spectroscopy, which was untargeted, this may have impacted our ability to detect metabolites at low concentrations (in our low volume cultures). Further studies using transcriptomic assays, LC-MS or GC-MS, in tandem with stable isotope labelling of HMOs could also be performed to probe these extra- vs. intracellular degradation cross-feeding questions [56].

In conclusion, this research provides new insight into how *Bifidobacterium* strains from the same infant have overlapping, but distinct HMO abilities (genomic, phenotypic, and putative cross-feeding). *Bifidobacterium* may therefore act as a foundation genus, acting within a community to maximise nutrient utilisation from breast milk, specifically HMOs. Determining these interactions with respect to infant diet, will be critical for the development of optimal multiple strain/species microbiota therapies to promote early-life health.

## Methods

### Bacterial isolation and strains

Faeces was collected from healthy (i.e., had not received any antibiotics/probiotics prior to sampling), full-term breast-fed infants in accordance with protocols laid out by the National Research Ethics Service approved UEA/QIB Biorepository (Licence no: 11208) and Quadram Institute Bioscience Ethics Committee (see Table S1). Infant faeces were isolated on RCM (Oxoid, Hampshire, UK) supplemented with mupirocin and l-cysteine (0.05 mg/mL each, Sigma-Aldrich, Dorset, UK). Bacterial isolates were randomly selected from agar plates, and all subsequent *Bifidobacterium* and *Lactobacillus* strains were grown at 37 °C in either RCM, de Man Rogosa and Sharpe (MRS) media, or modified MRS (mMRS) with specified carbohydrates in an anaerobic chamber (Don Whitley Scientific, Bingley, UK) containing 5% CO_2_, 10% hydrogen, 85% nitrogen gas.

### Bile salt survival and hydrolysis

To determine *Bifidobacterium* survival in bile, isolates were first grown in RCM and then subcultured using a 1:50 dilution into MRS ± 0.3% unfractionated bovine bile salt (Sigma-Aldrich), as described by [57]. After 48 h of stationary growth in an anaerobic chamber at 37 °C OD_600 nm_ using the Benchmark Plus microplate spectrophotometer (Bio-Rad) for both conditions. For the MRS plate the mean blank OD_600 nm_value was 0.1585 and for the anaerobic plate it was 0.1825. Data shown are mean values from three experimental repeats.To assess bile salt hydrolyase activity, overnight cultures were spotted (3 mL) onto MRS plates supplemented with l-cysteine and 0.5% w/v of either taurocholic acid, taurodeoxycholic acid, and sodium glycodeoxycholate bile salt (Sigma-Aldrich). Bile salt precipitation was assessed after a maximum of 96 h of anaerobic incubation at 37 °C. For both assays, uninoculated MRS media were used as a control.

### Aerotolerant assay

MRS media inoculated with a 1:50 dilution of each strain that had been grown aerobically or anaerobically at 37 °C for 48 h stationary. A blank average absorbance (from three wells per plate) was subtracted from each experimental OD_600_ reading, as described in [61]. For the aerobic plate the blank OD_600 nm_ value was 0.1507 and for the anaerobic plate it was 0.1549. Data shown are mean values from three experimental repeats.

### 16S rRNA gene library preparation and bioinformatics analysis

DNA extraction was performed using the FastDNA Spin Kit for Soil (MPBIO, California, USA) and V1–V2 16S rRNA gene primers as previously described [58]. Illumina MiSeq Raw reads underwent quality control using FASTX-Toolkit53 maintaining a minimum quality threshold of 33 for at least 50% of the bases. Passed read were then aligned against the SILVA database [59] using BLASTN55 [60] separately for both pairs. All output files were annotated using the paired-end protocol in MEGAN4 [61].

### Genomic DNA extraction

Bacteria was lysed with lysozyme, Proteinase K, RNase A (all from Roche Molecular Systems, West Sussex, UK), EDTA, and Sarkosyl NL30 (Sigma-Aldrich). Samples were purified with three rounds of phenol:chloroform:isoamyl alcohol (25:24:1; Sigma-Aldrich) extraction followed by multiple rounds of extractions with chloroform:isoamyl alcohol (24:1; Sigma-Aldrich). Genomic DNA pellets were resuspended in 10 mM Tris (pH8.0) and quantified using Qubit dsDNA BR assay kit (Invitrogen). See supplementary methods for additional information.

### Whole genome sequencing

DNA sequencing was performed using Illumina HiSeq 2500 platform with paired-end read length 2 × 125 bp, with an average coverage of 60×. Draft genome assemblies and annotation pipeline were performed as described in [62]. Publically assembled sequences (*n* = 64) were retrieved from NCBI Genomes database [63] and all genomes were annotated using Prokka v1.10 [64].

### Phylogenetic analysis of whole genomes

General feature format files of 83 *Bifidobacterium* strains were inputed into Roary pangenome pipeline v.3.8.0 to obtain core-genome data [65]. Phylogeny was reconstructed from core-genome alignment generated using MAFFT v7.305b [66] and cleaned up by manual curation and Gblocks [67, 68]. Maximum likelihood analysis was performed in Sea view v.4.0 [69] using PhyML v.3.1 with 100 bootstrap iterations [70]. Python3 module pyANI with default BLASTN+ settings was employed to calculate the ANI [71]. Species delineation cut-off was 95% identity.

### Functional annotation of genomes

For each genome, all ORFs were submitted to eggNOG-mapper for annotation and classification [72, 73]. Prediction of HMO clusters was performed by comparing known protein sequences to the draft genomes in this study using local BLAST (64) (e-value < 1e^−50^, percentage identify >70%). HMO clusters were annotated ‘present’ if over 90% of genes were homologous in the cluster. For prediction of GH, ORFs were submitted to the dbCAN web server [74] and the number of GH were calculated. Prophage presence was predicted using PHASTER [75, 76].

### HMO utilisation and cross-feeding

*Bifidobacterium* growth in mMRS + 2% (w/v) LNnT or 2′FL (Glycom, Hørsholm, Denmark) was determined using a microplate spectrophotometer. To assess cross-feeding potential within *Bifidobacterium* species we followed the experimental outline as described in [77], briefly we generated cell free supernatants (CFS) by sterile filtration of cultures grown anaerobically for 48 h in mMRS + 5% (w/v) LNnT or 2′FL. Fresh media were added to the CFS (1:1), and anaerobic growth was monitored every 15 min for 48 h in a microplate spectrophotometer (Tecan Infinite F50).

### ^1^H-nuclear magnetic resonance (NMR) spectroscopy analysis

For functional assessment of *Bifidobacterium* strains, media in which the bacterial cells had been grown were analysed using ^1^H-NMR spectroscopy. Media samples were mixed (2:1) with 0.2 M sodium phosphate buffer solution (pH 7.4) made in 100% deuterium oxide and 0.01% of sodium 3-(trimethylsilyl) [2,2,3,3,-2H4] propionate 3 mM NaN_3_. The mixture was vortexed and centrifuged and transferred to a 5 mm outer diameter NMR tube (Wilmad). One-dimensional spectroscopic data were acquired using a 500 MHz NMR spectrometer (Bruker Biospin, Germany) operating at 300 K. A standard one-dimensional NMR pulse sequence with water presaturation was applied to acquire spectroscopic data, using four dummy scans followed by 64 scans and collected into 24 K data points. ^1^H-NMR spectra were manually corrected for phase and baseline distortions and referenced to the TSP signal at *δ* 0.0, using the TopSpin 3.5 software package (Bruker Biospin, Germany). Spectra from the different bacterial strains grown under different conditions were overlaid in TopSpin and compared for differences. The integrate function was utilised to integrate peaks of interest. Spectral compound libraries (e.g., Human Metabolome DataBase, Biological Magnetic Resonance Data Bank) published literature and in-house spectral reference libraries were used to confirm metabolite assignments.

## Supplementary information

Supplementary methods

Figure S1

Figure S2

Figure S3

Figure S4

Figure S5

Figure S6

Figure S7

Figure S8

Figure S9

Table S1

Table S2

Table S3

Table S4

Table S5

Table S6

Table S7

Table S8

Table S9

## Data Availability

All metagenomic data are available at the European Nucleotide Archive, study accession ID PRJEB28188.
