# Supplementary material for: Breast milk-derived human milk oligosaccharides promote Bifidobacterium interactions within a single ecosystem
Source: ISME J. 2019 Nov 18;14(2):635–48. doi: 10.1038/s41396-019-0553-2 (PMC6976680; doi:10.1038/s41396-019-0553-2)
Supplement: Supplementary file 1 — Supplementary methods [file 41396_2019_553_MOESM1_ESM.docx]

**Supplementary methods**

**Bacterial isolation and strains.** Infant faces were collected from freshly soiled nappies and plated on RCM (Oxoid, Hampshire, UK) supplemented with 0.05mg/mL mupirocin and 0.05 mg/mL of L-cysteine (both from Sigma, Dorset, UK) within four hours of collection. All bifidobacteria isolates were grown at 37°C in RCM, de Man Rogosa and Sharpe (MRS) media or modified MRS (mMRS) with specified carbohydrates (including lactose and HMOs) as the main carbon source in an anaerobic chamber (miniMac, Don Whitley Scientific, Bingley, UK), unless otherwise specified. Control and type strains *B. breve* UCC2003, *B. breve* UCC2003DEPS, *B. pseudocatenulatum* DSM20438, *B. pseudocatenulatum* DSM20439, *B. longum* DSM20088, *B. breve* DSM20213, *B. infantis* DSM20219, *Lactobacillus casei* NCIMB4114, and *Escherichia coli* K-12 were also cultured as described above. All samples were collected according Quadram Institute Bioscience Ethics Committee, and sample collection was in accordance with protocols laid out by the National Research Ethics Service (NRES) approved UEA/QIB Biorepository (Licence no: 11208).

**16S rRNA gene library preparation and bioinformatics analysis.** DNA extraction was performed using the FastDNA Spin Kit for Soil (MPBIO, California, USA) following the manufacturer’s instructions, with the exception of extending the bead-beating step to 3 min. Faecal DNA concentration was measured using a Qubit® 2.0 fluorometer (Invitrogen, UK) and normalised to 5ng/mL prior to 16S rRNA gene library preparation. The V1-V2 region of the 16S rRNA gene was amplified using primers and conditions as in (Alcon-Giner C 2019). PCR library prep conditions included an initial 2 min denaturing step at 98°C, followed by 20 cycles of 98°C for 30s, 50°C for 30s, 72°C for 90s, and a final amplification step of 5 min at 72°C run in triplicate on a 96-well T-100 Thermocycler (Bio-Rad, Hertfordshire, UK). PCR products were pooled and purified using AMPure XP beads (Agencourt, Indianapolis, USA) and final quantification was assessed by PicoGreen prior running on the Illumina MiSeq platform with 300 bp paired end reads. Raw reads were initially processed through quality control using FASTX-Toolkit53 (<http://hannonlab.cshl.edu/fastx_toolkit/index.html>), maintaining a minimum quality threshold of 33 for at least 50% of the bases. Passed read were then aligned against the SILVA database (Quast et al 2013) (version: SILVA_132_SSURef_tax_silva) using BLASTN55 (Altschul et al 1997) (ncbi-blast-2.2.25+; Max e-value 10e-3) separately for both pairs. After performing the BLASTN alignment, all output files were annotated using the paired-end protocol in MEGAN bioinformatics software (Huson et al 2011). All raw files have been uploaded to the EBA (European Nucleotide Archive, <https://www.ebi.ac.uk/>) study accession ID PRJEB28188.

**Genomic DNA extraction.** Overnight bacterial cultures (10mL) were grown and used for phenol-chloroform DNA extraction. Briefly, bacterial pellets were re-suspended in 2 ml of 25% sucrose in 10 mM Tris and 1 mM EDTA at pH 8.0. Cells were then treated using 50 μl of 100 mg/ml lysozyme (Roche Molecular Systems, West Sussex, UK). Furthermore, 100 μl of 20 mg/ml Proteinase K (Roche Molecular Systems), 30 μl of 10 mg/ml RNase A (Roche Molecular Systems), 400 μl of 0.5 M EDTA (pH 8.0) and 250 μl of 10% Sarkosyl NL30 (Sigma-Aldrich) were added to the lysed bacterial suspension. The samples were then incubated on ice for 2 hours, and then placed in a 50 °C water bath overnight. The next day, we preformed three rounds of Phenol:Chloroform:Isoamyl Alcohol (25:24:1) (Sigma-Aldrich) extraction using Qiagen MaXtract High Density tubes (Qiagen, Manchester, UK). Followed by two rounds of extractions with Chloroform:Isoamyl Alcohol (24:1) (Sigma-Aldrich) to remove residual phenol before performing ethanol precipitation and wash (in 70% ethanol). Once dry, genomic DNA pellets were resuspended in 300 μl of 10 mM Tris (pH 8.0) and quantified using Qubit dsDNA BR assay kit. For qPCR Genomic DNA of *Bifidobacterium* strains was extracted from 1 ml culture with Fast DNA spin kit (MPbio, UK) following the manufacturer’s instructions. In brief, after enzymatic cell lysis, an additional mechanical lysis was performed in a 2-ml tube containing sterile zirconium beads (0.1mm in diameter) provided by the manufacturer and by running Fast prep instrument (mpbio, UK) for 3 cycles (speed of 6 for 1 minute and 5 minutes rest). The mechanically lysed cells were centrifuged at 14000 g for 15 min and the clear supernatant obtained was further processed as per manufacturers instructions to isolate genomic DNA without any loss.

**Whole genome sequencing.** Isolated DNA was subject to multiplex Sanger Illumina library preparation protocol followed by sequencing using Illumina HiSeq 2500 platform with read length 2 × 125 bp (paired-end reads) and an average sequencing coverage of 60×. Draft genome assemblies were generated using previously described assembly and annotation pipeline (Page et al 2016). Additionally, previously assembled publicly available sequences (n=64) were retrieved online from NCBI Genomes database. All genomes were annotated using Prokka v1.10 (Seemann 2014) (<https://github.com/tseemann/prokka>). All sequences were uploaded to the ENA under project number PRJEB28188.

**Co-culturing and qPCR experiments.** At t=0 equivalent concentrations (OD_600_ = 0.05) from each specified strain was added to mMRS media containing 2% of each HMO and monitored for growth by qPCR for 37 h. Cells were harvested at regular intervals and DNA isolated as described above. qPCR was carried out qPCR based on the fluorescent dye SYBR green I was performed by targeting species specific groEL gene as indicated in (Junick and Blaut 2012) on (Light Cycler, LC480, Roche). Each reaction mixture of 10 μl contained 1× QuantiFast SYBR green PCR Master Mix (HotStarTaq Plus DNA polymerase, QuantiFast SYBR green PCR buffer, dNTP mixture, and the ROX passive reference dye; Qiagen, UK), 250 nM each forward and reverse primer as indicated below, and 1 μl template DNA (<100ng). PCR cycling conditions: *B. longum* - 95°C for 5 min, 40 cycles at 94°C for 15 s, 65 °C for 15 s, 72°C for 15 s, and 83°C for 15 s; *B. pseudocatenulatum* - 95°C for 5 min, 40 cycles at 94°C for 15 s, 70 °C for 15 s, 72°C for 15 s, and 83°C for 15 s was used. The fluorescence of SYBR green I was measured after each amplification cycle and post-amplification melting-curve analysis was performed by slowly increasing the temperature from 68°C to 95°C (increments of 1%, holding for 10 s), while fluorescence was measured continuously. Thus, the specific PCR product was verified based on the specific melting temperature as indicated in table. Threshold cycles (CT) (the PCR cycle numbers at which the fluorescence exceeds the threshold above the calculated background) of less than 9 and more than 30 were excluded from the analysis. The concentration of species-specific DNA from each time point was calculated using a standard curve, which was generated by plotting the CT values obtained for 10-fold serial dilutions of the qPCR standards of known concentrations.

**Phylogenetic analysis of whole genomes.** General feature format files (GFF) of 83 *Bifidobacterium* strains were used as input for Roary pangenome pipeline v. 3.8.0 to obtain core-genome data (Page et al 2015). The phylogeny was reconstructed from the core-genome alignment generated using MAFFT v 7.305b (Katoh and Standley 2013) and subject to cleaning from poorly aligned positions using Gblocks (Castresana 2000, Talavera and Castresana 2007) (http://molevol.cmima.csic.es/castresana/Gblocks_server.html) and manual curation. Maximum likelihood analysis was performed in Seaview v. 4.0 (Gouy et al 2010) using PhyML v. 3.1 with 100 bootstrap iterations (Guindon et al 2010). Additionally, genomes of the isolates identified as *B. pseudocatenulatum* in infant individuals were subjected to the analysis with Roary, as described above, to obtain information about their core- and accessory-genome. Python 3 module pyANI with default BLASTN+ settings was employed to calculate the average nucleotide identity (ANI) between the 83 *Bifidobacterium* genomes (Pritchard et al 2016) (<https://github.com/widdowquinn/pyani>). A cut-off of 95% identity was used for species delineation.

**Functional annotation of genomes.** For each genome, all open reading frames were submitted to eggNOG-mapper (<http://eggnogdb.embl.de/#/app/emapper>) for annotation and classification (Huerta-Cepas et al 2016, Huerta-Cepas et al 2017). eggNOG-Mapper annotates each ORF and categorises it based on functional properties. The number of genes in each category was calculated and expressed as a proportion of all OFRs in that genome. A heat map of percentage ORFs in each category was generated using the heatmap.2 package in R. For genes unique to strains in V1 or V3, sequences from with LH9 (V1) or LH656 (V3) were submitted to eggNOG-mapper as above. Prediction of HMO clusters was performed by comparing the protein sequences for each gene in a cluster to the draft genomes described in this study using local BLAST (Altschul et al 1997) with an e-value cut off of 1e^-50^and percentage identify of 70%. A heat map of the presence or absence of each cluster was generated using the heatmap.2 package in R. A cluster was deemed to be present if more a genome contains homologues of over 90% of genes in the cluster. A cluster was annotated as partially present if genes involved in the enzymatic digestion of HMOs, but not those involved in transport, were present and if no genes were identified then the cluster was annotated as absent.

**Prediction of GH.** For each genomes all ORFs were submitted to the dbCAN web server (<http://csbl.bmb.uga.edu/dbCAN/>) (Yin et al 2012). The dbCAN database uses data from the CAZy database ([http://www.cazy.org](http://www.cazy.org/)) to build HMM models of each GH family and applies these model to predict the presence of GH from submitted protein coding sequences. The percentage GH as a function of total CDS was calculated for each genome and a heatmap generated using the heatmap.2 package in R.

**Prediction of prophages.** The draft genomes of LH13 and LH656 were submitted to PHASTER (http://phaster.ca/) to predict the presence of prophage genes in the genome (Arndt et al 2016, Zhou et al 2011). The genomic location of these predicted prophage regions was compared to unique genes identified by ROARY.

**Bile salt survival and hydrolysis**. To determine *Bifidobacterium* survival in bile, isolates were first grown in RCM and then subcultured using a 1:50 dilution into MRS ± 0.3% unfractionated bovine bile salt (Sigma-Aldrich), as described by (Fanning et al 2012). After 48h of stationary growth in an anaerobic chamber at 37°C OD_600nm_ using the Benchmark Plus microplate spectrophotometer (Bio-Rad) for both conditions. For the MRS plate the mean blank OD_600nm_value was 0.1585 and for the anaerobic plate it was 0.1825. Data shown is mean values from three experimental repeats. To assess bile salt hydrolyase activity, overnight cultures were spotted (3mL) onto MRS plates supplemented with L-cysteine and 0.5% w/v of either taurocholic acid, taurodeoxycholic acid, and sodium glycodeoxycholate bile salt (Sigma-Aldrich). Bile salt precipitation was assessed after a maximum of 96 hours of anaerobic incubation at 37°C. For both assays, uninoculated MRS media was used as a control.

**Aerotolerant assay.** MRS media inoculated with a 1:50 dilution of each strain that had been grown aerobically or anaerobically at 37°C for 48 hours stationary. The average absorbance of un-inoculated MRS media was based on was subtracted from all OD_600nm_ readings. For the aerobic plate the blank OD_600nm_ value was 0.1507 and for the anaerobic plate it was 0.1549. Data shown is mean values from three experimental repeats.

**HMO utilisation and cross-feeding.** Growth kinetics of the 19 novel *Bifidobacterium* isolates and control strains using either LNnT or 2’FL (Glycom, Hørsholm, Denmark) as the soul carbon source (2%w/v) in mMRS was determined using a microplate spectrophotometer. For cross-feeding experiments, HMO-users were grown in mMRS and LNnT or 2’FL in an excess of 5%w/v, to ensure the carbon source was not the limiting factor for overnight growth. Cultures were then centrifuged at 4°C for 10 minutes at 4,000 rpm and the supernatant was sterile filtered (0.22mm filter) twice, prior to being used for growth with strains that had been identified to be unable to degrade the HMOs. Spent media was then added in a 1:1 ratio with mMRS media and then inoculated with the specified strains and monitored for growth by a microplate spectrophotometer (Tecan Infinite F50) with agitation every 15 minutes for 48 hours.

**^1^H-Nuclear Magnetic Resonance (NMR) Spectroscopy analysis.** For functional assessment of *Bifidobacterium* strains, media in which the bacterial cells had been grown, were analysed using ^1^H-NMR Spectroscopy. Media samples were prepared for analysis, by transferring 400μL of cell media into a microtube containing 200μL of 0.2M sodium phosphate buffer solution (pH 7.4) made in 100% deuterium oxide (which was needed for the field lock of the NMR spectrometer). The buffer solution also contained 0.01% of sodium 3-(trimethylsilyl) [2,2,3,3,-2H4] propionate (TSP) as an internal reference standard for calibration of acquired spectral profiles, and 3mM NaN3 as a preservative. The mixture was vortexed and centrifuged for 10 seconds followed by transfer into a 5mm outer diameter NMR tube (Wilmad). One-dimensional spectroscopic data were acquired using a 500 MHz NMR spectrometer (Bruker Biospin, Germany) operating at 300 K. A standard one-dimensional NMR pulse sequence with water pre-saturation was applied to acquire spectroscopic data, using 4 dummy scans followed by 64 scans and collected into 24 K data points. ^1^H NMR spectra were manually corrected for phase and baseline distortions and referenced to the TSP signal at *δ* 0.0, using the TopSpin 3.5 software package (Bruker Biospin, Germany). Spectra from the different bacterial strains grown under different conditions were overlaid in TopSpin and compared for differences. The integrate function was utilised to integrate peaks of interest. Spectral compound libraries (e.g. Human Metabolome DataBase (HMDB), Biological Magnetic Resonance Data Bank) published literature and in-house spectral reference libraries were used to confirm metabolite assignments.

**References**

Alcon-Giner C Dalby M, Caim S, Ketskemety J, Shaw A, Sim K, *et al*. (2019). Microbiota supplementation with Bifidobacterium and Lactobacillus modifies the preterm infant gut microbiota and metabolome. *[Preprint] bioRxiv* **doi.org/10.1101/698092**.

Altschul SF, Madden TL, Schaffer AA, Zhang J, Zhang Z, Miller W *et al* (1997). Gapped BLAST and PSI-BLAST: a new generation of protein database search programs. *Nucleic Acids Res* **25:** 3389-3402.

Arndt D, Grant JR, Marcu A, Sajed T, Pon A, Liang Y *et al* (2016). PHASTER: a better, faster version of the PHAST phage search tool. *Nucleic Acids Res* **44:** W16-21.

Castresana J (2000). Selection of conserved blocks from multiple alignments for their use in phylogenetic analysis. *Mol Biol Evol* **17:** 540-552.

Fanning S, Hall LJ, Cronin M, Zomer A, MacSharry J, Goulding D *et al* (2012). Bifidobacterial surface-exopolysaccharide facilitates commensal-host interaction through immune modulation and pathogen protection. *Proc Natl Acad Sci U S A* **109:** 2108-2113.

Gouy M, Guindon S, Gascuel O (2010). SeaView version 4: A multiplatform graphical user interface for sequence alignment and phylogenetic tree building. *Mol Biol Evol* **27:** 221-224.

Guindon S, Dufayard JF, Lefort V, Anisimova M, Hordijk W, Gascuel O (2010). New algorithms and methods to estimate maximum-likelihood phylogenies: assessing the performance of PhyML 3.0. *Syst Biol* **59:** 307-321.

Huerta-Cepas J, Szklarczyk D, Forslund K, Cook H, Heller D, Walter MC *et al* (2016). eggNOG 4.5: a hierarchical orthology framework with improved functional annotations for eukaryotic, prokaryotic and viral sequences. *Nucleic Acids Res* **44:** D286-293.

Huerta-Cepas J, Forslund K, Coelho LP, Szklarczyk D, Jensen LJ, von Mering C *et al* (2017). Fast Genome-Wide Functional Annotation through Orthology Assignment by eggNOG-Mapper. *Mol Biol Evol* **34:** 2115-2122.

Huson DH, Mitra S, Ruscheweyh HJ, Weber N, Schuster SC (2011). Integrative analysis of environmental sequences using MEGAN4. *Genome Res* **21:** 1552-1560.

Junick J, Blaut M (2012). Quantification of human fecal bifidobacterium species by use of quantitative real-time PCR analysis targeting the groEL gene. *Appl Environ Microbiol* **78:** 2613-2622.

Katoh K, Standley DM (2013). MAFFT multiple sequence alignment software version 7: improvements in performance and usability. *Mol Biol Evol* **30:** 772-780.

Page AJ, Cummins CA, Hunt M, Wong VK, Reuter S, Holden MT *et al* (2015). Roary: rapid large-scale prokaryote pan genome analysis. *Bioinformatics* **31:** 3691-3693.

Page AJ, De Silva N, Hunt M, Quail MA, Parkhill J, Harris SR *et al* (2016). Robust high-throughput prokaryote de novo assembly and improvement pipeline for Illumina data. *Microb Genom* **2:** e000083.

Pritchard L, Glover RH, Humphris S, Elphinstone JG, Toth IK (2016). Genomics and taxonomy in diagnostics for food security: soft-rotting enterobacterial plant pathogens. *Anal Methods-Uk* **8:** 12-24.

Quast C, Pruesse E, Yilmaz P, Gerken J, Schweer T, Yarza P *et al* (2013). The SILVA ribosomal RNA gene database project: improved data processing and web-based tools. *Nucleic Acids Res* **41:** D590-596.

Seemann T (2014). Prokka: rapid prokaryotic genome annotation. *Bioinformatics* **30:** 2068-2069.

Talavera G, Castresana J (2007). Improvement of phylogenies after removing divergent and ambiguously aligned blocks from protein sequence alignments. *Syst Biol* **56:** 564-577.

Yin Y, Mao X, Yang J, Chen X, Mao F, Xu Y (2012). dbCAN: a web resource for automated carbohydrate-active enzyme annotation. *Nucleic Acids Res* **40:** W445-451.

Zhou Y, Liang Y, Lynch KH, Dennis JJ, Wishart DS (2011). PHAST: a fast phage search tool. *Nucleic Acids Res* **39:** W347-352.
