# Supplementary material for: Breast milk-derived human milk oligosaccharides promote Bifidobacterium interactions within a single ecosystem
Source: ISME J. 2019 Nov 18;14(2):635–48. doi: 10.1038/s41396-019-0553-2 (PMC6976680; doi:10.1038/s41396-019-0553-2)
Supplement: Supplementary file 2 — Figure S1 [file 41396_2019_553_MOESM2_ESM.pdf]

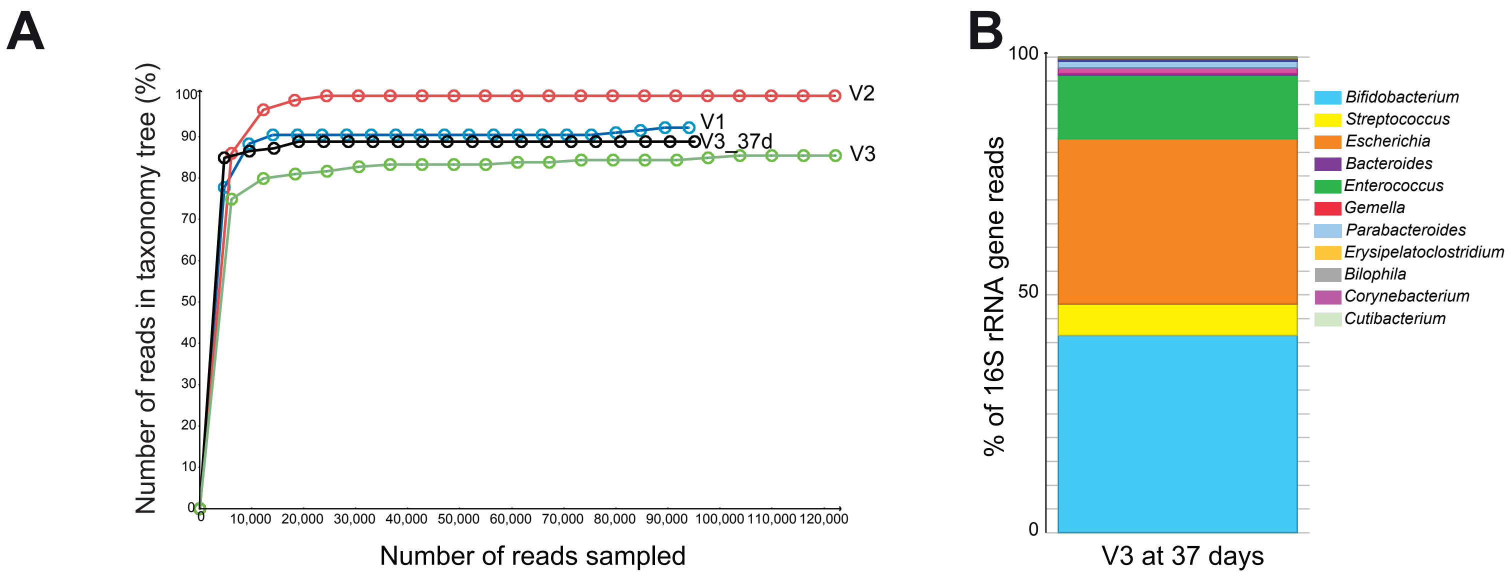

Supplementary figure 1. (A) taxonomy refraction curves from 16S rDNA data from infant fecal sample from infant V1, V2, V3 at both 37 and 159 days. (B) 16S rDNA profile of top 11 Genus identified in infant V3 at 37 days faecal sample shown as a percentage of reads and analysed by MEGAN.
