# Supplementary material for: Breast milk-derived human milk oligosaccharides promote Bifidobacterium interactions within a single ecosystem
Source: ISME J. 2019 Nov 18;14(2):635–48. doi: 10.1038/s41396-019-0553-2 (PMC6976680; doi:10.1038/s41396-019-0553-2)
Supplement: Supplementary file 3 — Figure S2 [file 41396_2019_553_MOESM3_ESM.pdf]

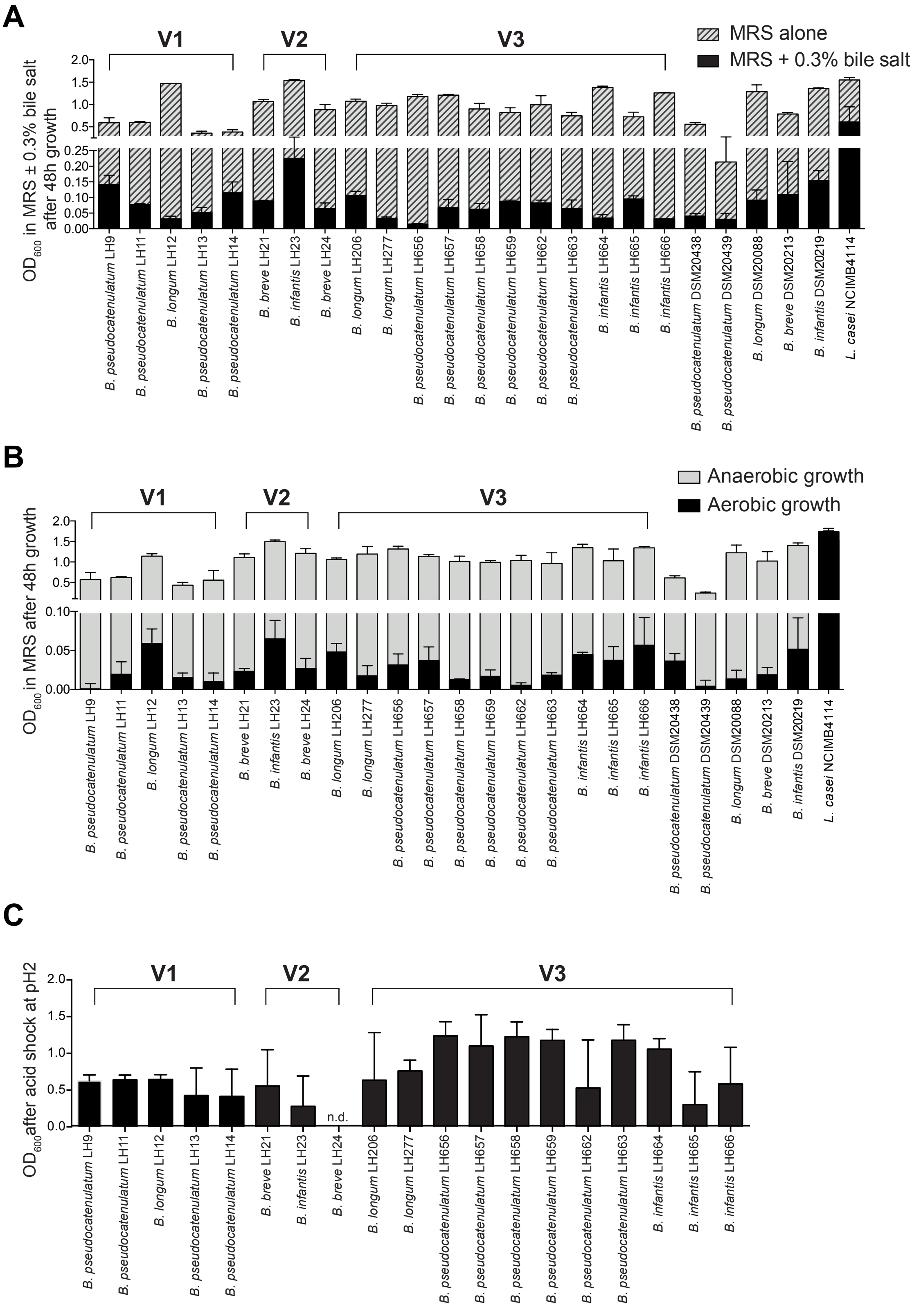

Supplementary figure 2: Bifidobacterial survival after exposure to intestinal environmental stressors. (A) Bacterial survival in MRS supplemented with 0.3% bile salts was measured after 48h growth and compared to parallel cultures grown in MRS anaerobically at 37˚C. Data shown from five independent experiments; n.d. denotes measurement not detectable. Blank/media only values are subtracted from all points in each experiment, all negative values are represented as zero. Isolates from Baby V1 are represented as black bars, V2 are grey bars, V3 are dotted bars, and the tested type and control strains are shown as white bars. Error bars represent SEM. (B) Bacterial growth in MRS was measured for each strain after 30h growth in an aerobic environment and compared to parallel samples grown anaerobically. Measurements shown are representative of three independent experimental repeats. (C) Ability to withstand acid shock for 4 h at a pH2. Following acid shock, strains were incubated for 48 h in an anaerobic cabinet at 37˚C. Growth (optical density) was then measured. Data shown is mean ± standard deviation for 3 independent experiments
