# Supplementary material for: Breast milk-derived human milk oligosaccharides promote Bifidobacterium interactions within a single ecosystem
Source: ISME J. 2019 Nov 18;14(2):635–48. doi: 10.1038/s41396-019-0553-2 (PMC6976680; doi:10.1038/s41396-019-0553-2)
Supplement: Supplementary file 4 — Figure S3 [file 41396_2019_553_MOESM4_ESM.pdf]

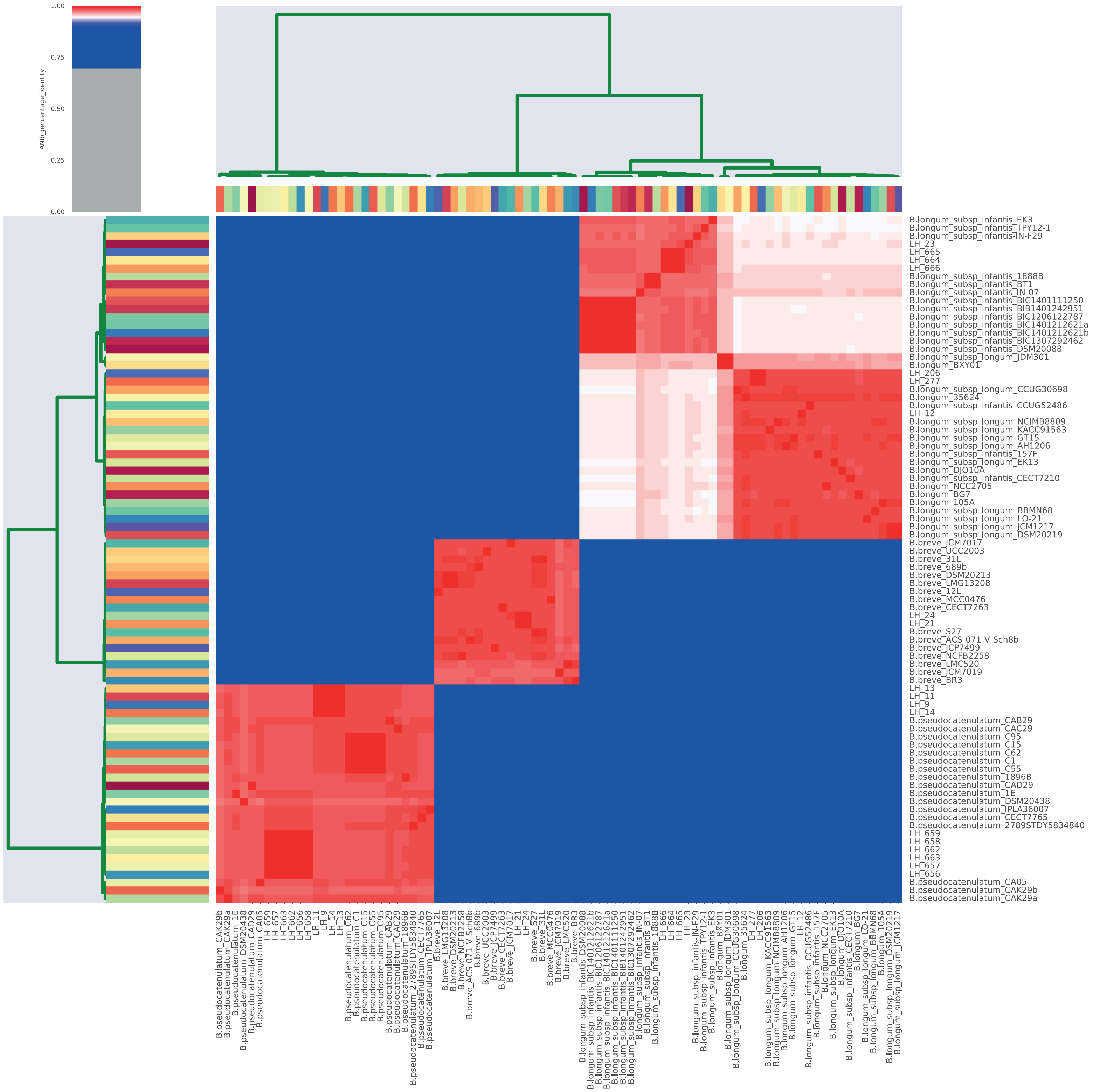

Supplementary figure 3: Average Nucleotide Identity (ANI) between 83 *Bifidobacterium* strains. Genetic relatedness above 95% identity (white and red) is used as a cut-off for species delineation.
