# Supplementary material for: Breast milk-derived human milk oligosaccharides promote Bifidobacterium interactions within a single ecosystem
Source: ISME J. 2019 Nov 18;14(2):635–48. doi: 10.1038/s41396-019-0553-2 (PMC6976680; doi:10.1038/s41396-019-0553-2)
Supplement: Supplementary file 6 — Figure S5 [file 41396_2019_553_MOESM6_ESM.pdf]

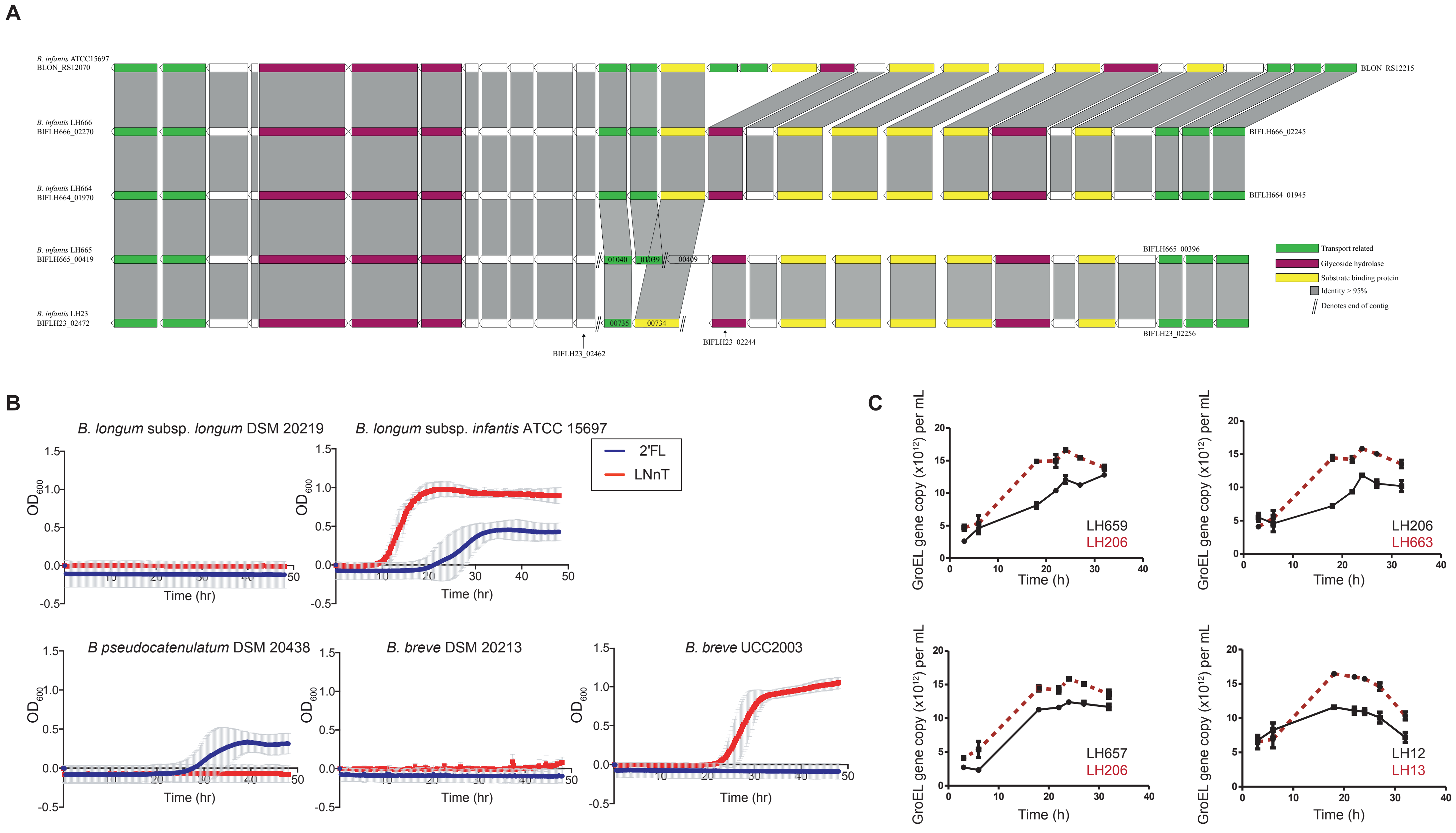

Supplementary figure 5: (A) Genomic arrangement of HMO utilisation cluster found in *B. infantis* strains ATCC 15697, LH664, LH665, LH666, and LH23. (B) Growth kinetics of type strains in mMRS with either HMO 2'FL (blue) or LNnT (red) as a sole carbon source. Data shown is a representative graph of three independent experimental repeats (mean ± standard deviation), (C) Quantification of DNA concentration from strains grown during co-culturing of HMO metabolising (red line) and cross-feeding (black line) strains. qPCR was performed on extracted DNA and the number of species specific groEL copies per ml of DNA calculated and plotted as an indication of cell number.
