# Supplementary material for: Breast milk-derived human milk oligosaccharides promote Bifidobacterium interactions within a single ecosystem
Source: ISME J. 2019 Nov 18;14(2):635–48. doi: 10.1038/s41396-019-0553-2 (PMC6976680; doi:10.1038/s41396-019-0553-2)
Supplement: Supplementary file 7 — Figure S6 [file 41396_2019_553_MOESM7_ESM.pdf]

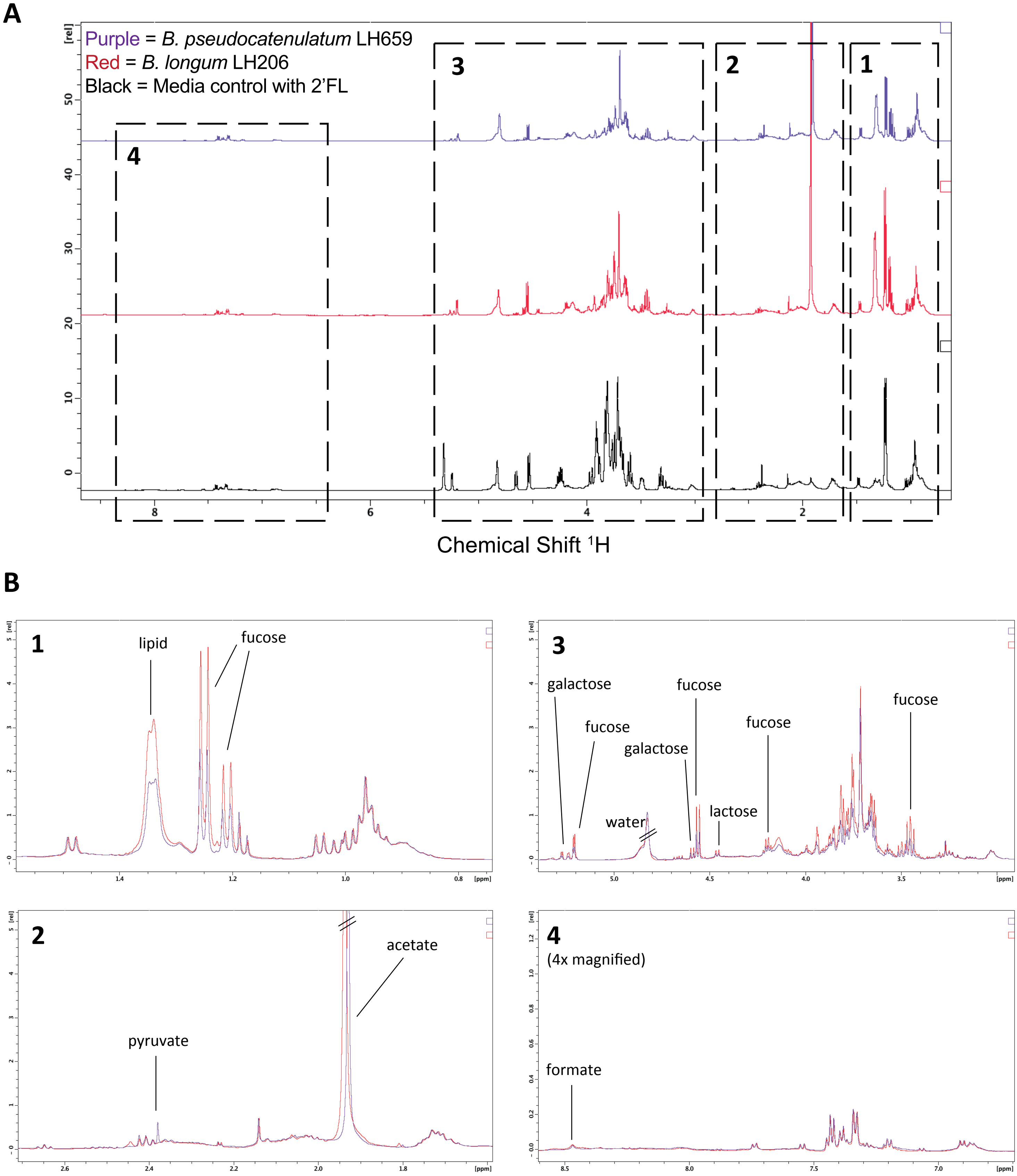

Supplementary figure 6: (A)Whole 1H NMR spectrum for 2'FL cross-feeding experiment between Red = *B. longum* LH206 (HMO degrader), Purple = *B. pseudocatenulatum* LH659, and media control supplemented with 2% w/v 2'FL (black) (B) Enlarged overlapping spectrums identified in (A) of metabolites identified after *B. longum* LH206 growth, and the subsequent growth of *B. pseudocatenulatum* LH659 on the conditioned media with annotated metabolites.
